# Seasonal Responses of Hydraulic Function and Carbon Dynamics in Spruce Seedlings to Continuous Drought

**DOI:** 10.3389/fpls.2022.868108

**Published:** 2022-05-04

**Authors:** Yangang Han, Jiaojiao Deng, Wangming Zhou, Qing-Wei Wang, Dapao Yu

**Affiliations:** ^1^CAS Key Laboratory of Forest Ecology and Management, Institute of Applied Ecology, Chinese Academy of Sciences, Shenyang, China; ^2^University of Chinese Academy of Sciences, Beijing, China

**Keywords:** northern temperate forests, drought, mortality, C starvation, dormant season, growing season, *Picea jezoensis*

## Abstract

Drought is expected to increase in the frequency and duration associated with climate change. Although hydraulic function and carbon (C) storage have been widely recognized as key components to plant survival under a single drought, the physiological responses to continuous drought remain largely unknown, particularly for high northern temperate and boreal forests which are sensitive to water stress. In this study, we quantified the survival, growth, gas exchange, water relations, and nonstructural carbohydrates (NSCs) in 3-year-old Jezo spruce (*Picea jezoensis*) seedlings responding to continuous drought stress. Seedlings were maintained in drought conditions for 392 days, covering two growing and one dormant winter season. Seedlings subjected to drought showed a significant decrease in net photosynthesis rate (*A*_*net*_) and stomatal conductance (*g*_*s*_) in both growing seasons, and biomass in the second growing season. The seedling mortality continuously increased to 35.6% at the experimental end. Notably, responses of C storage and leaf water potential to drought varied greatly depending on seasons. Living seedlings exposed to drought and control treatments had similar NSC concentrations in both growing seasons. However, seedlings with concentrations of both the soluble sugars and starch less than 1% in root died in the winter dormant season. In the second growing season, compared with the control treatment, droughted seedlings had significantly lower leaf water potential and stem wood-specific hydraulic conductivity (*K*_w_). Meanwhile, the leaf predawn water potential did not recover overnight. These suggest that C starvation might be an important reason for seedlings that died in the winter dormant season, while in the growing season drought may limit seedling survival and growth through inducing hydraulic failure. Such seasonal dependence in hydraulic dysfunction and C depletion may lead to higher mortality in spruce forests facing extended drought duration expected in the future.

## Introduction

Climate change has been leading to frequent and continuous drought globally (Dai, 2013). Extreme drought events could increase the massive tree mortality, especially in the temperate and boreal regions, where forests are much sensitive to changes in water conditions (Allen et al., 2015). For instance, the 2011 unprecedented drought induced the mortality of more than 300 million trees in the USA (Yan et al., 2022). The severe drought and massive tree mortality would compromise forest ecosystems, the regional ecological security, and the terrestrial carbon (C) sink (McDowell et al., 2020). Thus, understanding the response and adaptation mechanism of trees to continuous drought is crucial to predicting how forest ecosystems and the C-cycle feedbacks respond to climate change (Choat et al., 2018).

Empirical evidence has shown that hydraulic failure is the primary reason for tree mortality induced by drought (Adams et al., 2017), which is resulted from the irreversible dysfunction in xylem water transportation (McDowell et al., 2008). However, tree mortality under continuous drought may be caused by both water and C relation which are interdependent inside trees (McDowell et al., 2008). Continuous drought could not only inhibit tree water transport, leading to severe xylem hydraulic dysfunction before death (López et al., 2021) but also hinder photosynthetic function by inducing embolism in the vascular conduits, consequently reducing nonstructural carbohydrates (NSCs) reserve (Ivanov et al., 2019). In turn, C depletion causes less support for the refilling of the embolisms which is essential for the repair of hydraulic function (Secchi and Zwieniecki, 2011; Tomasella et al., 2019, 2021). The complex relationship between water and C confuses understanding of mechanisms of drought-induced mortality (Gessler et al., 2018). Furthermore, previous studies also reported that these two mechanisms occurred in the same species accounting for tree death, e.g., *Pinus edulis* (Sevanto et al., 2014), while the occurrence timing may depend on the drought stage (Kono et al., 2019). These suggest that the causes of drought-induced mortality vary with drought duration (Mitchell et al., 2013; Kono et al., 2019).

In temperate forests, variation in the season and duration of drought differently affects hydraulic and C dynamics in trees (Gebauer et al., 2020; Charrier et al., 2021). In summer (growing seasons), trees require more water for transpiration and photosynthesis due to relatively high temperatures and active physiological activities (Morales et al., 2021). It leads trees to face a high risk of hydraulic failure (Nardini et al., 2013), accompanied by C depletion under drought conditions during the growing season (McDowell et al., 2008). In winter (dormant seasons), the C reserve is critical for tree survival rather than water transportation because C reserve plays a key role in cold and frost resistance (Charrier et al., 2021), while severe embolism (with percentage loss of conductance (PLC) closed to 100%) is not lethal due to the low transpiration and cessation of water absorption (Christensen-Dalsgaard and Tyree, 2014; Maruta et al., 2020; Mayr et al., 2020). These findings suggest that mechanisms of drought-induced tree mortality interact with seasonality in temperate forest ecosystems. In addition, C accumulation in trees only occurs in the growing season, which is critical for the survival and regrowth of trees in the following seasons (Tixier et al., 2019; D'Andrea et al., 2021). However, most of the studies generally focus on the response of hydraulic and C storage to drought conducted in growing seasons. Up to now, how hydraulic and C storage in trees respond to continuous drought stress across seasons still remains poorly understood (Galvez et al., 2013).

Spruce (*Picea spp*.) is widely distributed in northern temperate and boreal forests (Brenzel, 2001), and is more sensitive to water stress than other conifer species (Kharuk et al., 2015). Drought has been generally considered as a driver to spruce mortality occurring in the world (Schuldt et al., 2020; Obladen et al., 2021). In the last two decades, considerable mortality of Jezo spruce (*Picea jezoensis*) was also observed in Changbai Mountains Natural Reserve (CMNR) in northeast China, the largest primitive temperate forest reserve at the same latitude in the world. The tree-ring data showed that Jezo spruce might suffer from the warming-induced water deficit in the early and late growing season (Yu et al., 2021). To clarify the internal physiological mechanisms, we conducted a 392-day drought manipulation experiment on 3-year-old Jezo spruce seedlings and quantified seedling survival, growth, gas exchange, water relations, and C storage and dynamics responding to continuous drought over two growing and one winter dormant seasons. We explored the following questions: (1) How do the gas exchange, growth, and C and hydraulic of Jezo spruce seedlings respond to continuous drought? (2) Whether the cause of drought-induced mortality of Jezo spruce seedlings varies between seasons during continuous drought in temperate forests?

## Materials and Methods

### Population Distribution Area

The spruce-fir forest, naturally distributing between 1,100 and 1,800 m a.s.l. (above sea level), is one of the main forest types in CMNR in the north temperate climate zone of eastern Eurasia (41°31′-42°28′ N and 127°09′-128°55′ E). The climate here is classified as the temperate continental monsoon climate with the characteristics of warm and rainy summer, and cold and dry winter. The monthly mean temperature ranges between −17.5°C in January and 20.1°C in July. The mean annual precipitation is 680 mm, with 80% of precipitation in the growing season (Yu et al., 2011, 2013).

### Experimental Design

In April 2019, we established the experiment at the Changbai Mountain Forest Ecosystem Research Station, Chinese Academy of Sciences. The ambient climate data in the station were shown in Supplementary Figure S1.

Three-year-old Jezo spruce seedlings were collected from a local nursery garden and planted into 31 wooden rectangle containers (length × width × height: 120 × 25 × 25 cm) with 7 individuals per container on 30th April 2019. Wooden rectangle containers were placed under a transparent rain roof in a forest gap, avoiding rapid water loss due to strong sunlight exposure. The light condition was similar in different positions under the roof according to condition premeasurement. The soil used in the experiment was obtained from local forests' topsoil without the large stone and roots. Since 5th July 2019, 132 healthy seedlings in 19 containers were not watered throughout the experiment as the drought treatment, while water was continually supplied to the amount of 84 homogeneous seedlings in 12 containers in the control treatment (see the initial seedling status in Table 1). The significant difference in the size (stem base diameter and height) between control and drought was not detected (*p* > 0.05).

**Table 1:** The seedlings' size before the experiment. Values were mean ± SE. The difference in the size between control and drought was not significant (*p* > 0.05).

**Treatment**	**Stem base diameter/mm**	**Height/cm**
Control (*n* = 84)	3.95 ± 0.67	20.25 ± 3.2
Drought (*n* = 132)	3.98 ± 0.59	21.20 ± 2.81

The experiment period was from 5th July 2019 to 30th July 2020 (392 days in total), including the first growing season (GS2019: D1–D88, 5th July to 30th September), winter dormant season (DP2019: D89–D180, 1st October to 31st December; DP2020: D181–D301, 1st January to 30th April), and the second growing season (GS2020: D302–D392, 1st May to 31st July) referring to Yu et al. (2021). In the dormant season, irrigation was stopped as the stomatal closure and transpiration stopped, referring to previous studies (Fajstavr et al., 2019).

During the drought process, the soil water content was measured with the gravimetric method. The soil water content was determined as the ratio of water weight to the soil sample weight (Piper and Fajardo, 2016). The soil water content was not measured in the winter dormant season due to low evapotranspiration and soil frost in the study area (Yang et al., 2006).

According to the phenology, we harvested seedlings on the D49 (August 2019, the middle growing season), D127 (November 2019, the early dormant period), D270 (March 2020, the late dormant period), and D392 (July 2020, the middle growing season). For each harvest, one container of each treatment was randomly selected, and all living seedlings in that container were sampled and then transported to the laboratory in a cooling box. Collected seedlings were divided into leaves, branches (including stems and twigs), and roots. Samples were dried at 105°C for 30 min to stop the enzymatic activity, then oven-dried at 65°C for 48 h, and finally stored at −20°C for further NSC analyses (Huang et al., 2019).

### Measurement of Seedling Mortality

To evaluate the effects of drought on seedlings' survival, we examined seedling mortality weekly during the growing season and biweekly during the dormant season. It is difficult to determine the exact death time of the evergreen species. In this study, dead seedlings were identified when all leaves turned yellow and fallen (referring to previous studies, e.g., Hartmann et al., 2013; Ivanov et al., 2019). The mortality rate of seedlings was counted during the experimental period. In this work, we calculated the mortality rate on the D49, D127, D350, D372, and D392 as:


(1) $$\begin{array}{r} \text{Cumulative mortality rate }\left(\text{\%}\right)\;=\;100\;\times \;\sum n_{i}\;/\;\text{N} \end{array}$$

where *i* is the days after the treatment; *n*_*i*_ is the number of dead seedlings from D_1_ to *D*_*i*_; and *N* is the total number of seedlings of each treatment at the beginning of the experiment.

### Measurement of Leaf Water Potential

Leaf predawn water potential (ψ_pd_) between 3:00 and 4:00 and middy water potential (ψ_md_) between 12:00 and 13:00 from 4 to 7 living seedlings were measured on sunny days (D17, D47, D349, D370, and D390) using a pressure chamber (PMS1000; Albany, OR, USA; maximum measurement: 8 MPa). One twig from each seedling was selected for the measurement. No repeated measurement was conducted on the same seedling throughout the experiment.

### Measurement of Stem Hydraulic Conductivity

The hydraulic conductivity was measured on the same seedlings that were used for leaf water potential measurement at D350, D371, and D391, respectively. The seedlings were cut at the stem base in the next early morning (before sunrise) after leaf water potential measurement. Stems were cut immediately under water to avoid the formation of embolism during sampling, and were transported to the laboratory (<100 m). In the laboratory, the stem segment was recut repeatedly under water at two ends to release the tension. The segment with 7 cm length was used to measure native hydraulic conductivity (*K*_h_, kg m s^−1^ MPa^−1^). The *K*_h_ was measured using a pressure induced by the gravity of a hydraulic head of 50 cm with the 20 mM KCl solution. The *K*_h_ was calculated by: *K*_h_=*J*_v_ / (*F* / *L*), where *J*_v_ is the flow rate (kg s^−1^), *F* is the gravity-induced driving pressure (MPa), and *L* is the length of the segment (m). The *K*_h_ was divided by the xylem wood area to calculate the stem wood-specific hydraulic conductivity (*K*_w_, kg m^−1^ s^−1^ MPa^−1^) (Fang et al., 2018).

### Measurement of Net Photosynthesis Rate and Stomatal Conductance

Net photosynthesis rate (*A*_*net*_) and stomatal conductance (*g*_*s*_) were measured between 9:00 and 12:00 on sunny days (D17, D47, D349, D370, and D390). The measurements were done under the ambient light (*c*. 1,000 μmol m^−2^ s^−1^) and CO_2_ concentration (*c*. 400 μmol mol^−1^). The relative difference in *A*_*net*_ and *g*_*s*_ between drought and control was calculated: Relative to control (%) =100 × (Value_drought_ / Mean_control_), where the Value_drought_ is the value of drought seedling and Mean_control_ is the mean value of control at the same time.

### Nonstructural Carbohydrates (NSCs) Quantification

Nonstructural carbohydrate (NSC) concentrations (soluble sugars + starch) were quantified according to the standardized protocols by Landhausser et al. (2018). For soluble sugars extraction, approximately 30 mg sample was bathed at 90°C for 10 min after mixing with 1.5 ml 80% (v/v) ethanol. The mixture was centrifuged at 3,500 rpm for 10 min, and the supernatant was used for soluble sugars quantification. For soluble sugars quantification, the solution was colored with phenol-sulfuric and the absorbance was determined at 490 nm with a microplate reader [Multiskan Sky, Thermofisher Scientific (China) Co., Ltd.]. For starch digestion, the pellet after soluble sugars extraction was digested at 85°C for 1 h with the α-amylase from *Aspergillus oryzae* (600 U/ml, Macklin A861434). The supernatant obtained after centrifuging was further digested with amyloglucosidase from *Aspergillus niger* (100 U/ml, Macklin A800618). For starch quantification, the solution color was regulated by peroxidase-glucose oxidase, and the absorbance was determined at 525 nm. Concentrations of soluble sugars, starch, and NSC were expressed as a percentage of dry matter (% d.w.).

### Statistical Analysis

Two-way ANOVA was used to analyze the effects of season, drought, and their interaction on concentrations of NSC, soluble sugars, and starch. The difference in variables among control, drought, and dead seedlings was assessed using least significant difference (LSD) with package “agricolae” (Mendiburu, 2021). The difference in soil water content, *A*_*net*_, and *g*_*s*_ between control and drought seedlings was evaluated using Wilcoxon rank-sum test with the package “stats” (R Core Team, 2020). In addition, the difference between ψ_pd_ and ψ_md_ at the same date was evaluated by Wilcoxon rank-sum test. The starch concentration in the root and the *K*_w_ were logarithmic (ln) transformed to meet normality and homogeneity before the analysis. Statistical analysis for all data was conducted in R 4.0.0 (R Core Team, 2020).

## Results

### Response of Seedling Mortality Rate to Continuous Drought

**Figure 1:**
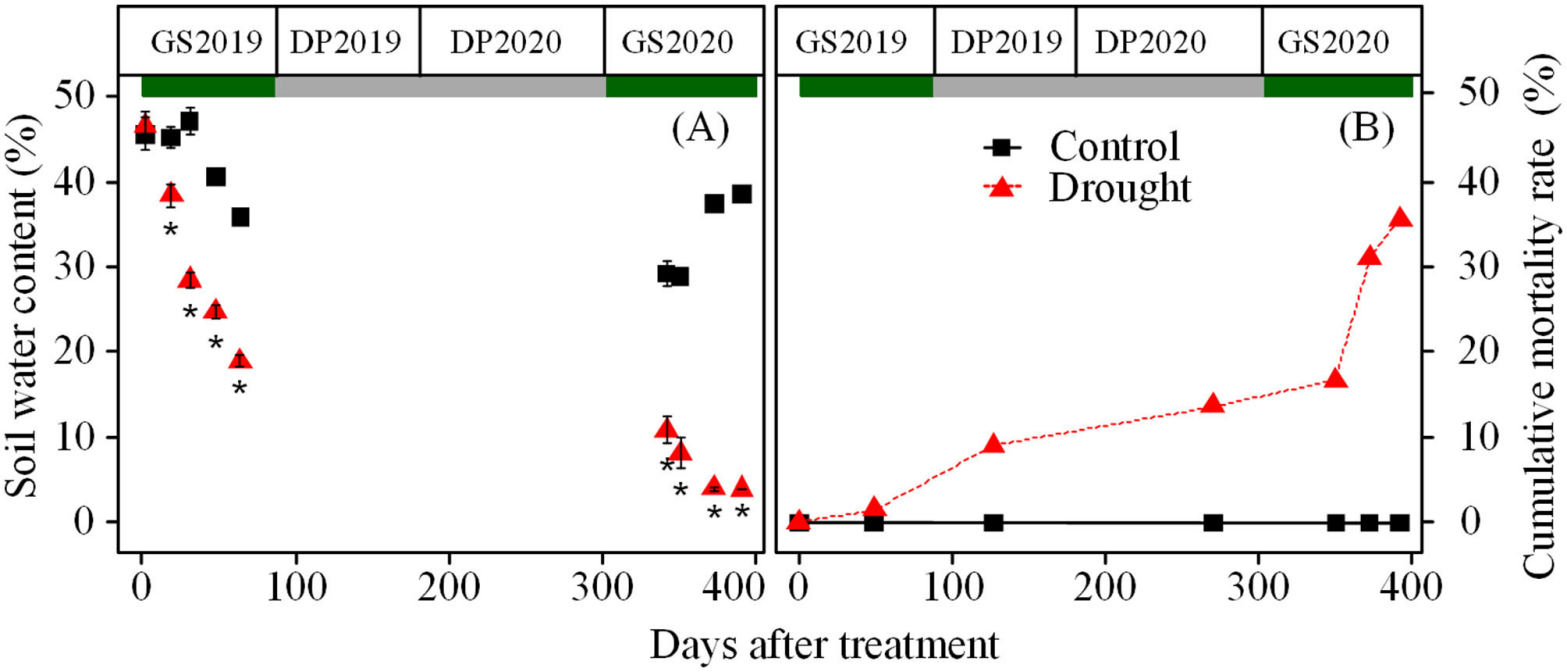
Variation in soil water content **(A)**, and cumulative mortality rate **(B)** over the experimental period. Green and gray rectangles indicate growing and dormant seasons, respectively. GS2019, the first growing season (D1–D88); DP2019 and DP2020, the early and late winter dormant seasons (D89–D180 and D181–D301); and GS2020, the second growing season (D302–D392). Black square and red triangle represent control and drought treatments, respectively. Asterisk indicates a significant difference (*p* < 0.05) in soil water content between control and drought treatments, analyzed using Wilcoxon rank-sum test. Values are mean ± SE (*n* = 4–6) **(A)**.

Soil water content in control ranged from 47.1 to 35.8% in the GS2019 and stabled at 38% in the GS2020, but in drought condition rapidly decreased from 47.1 to 19.0% in GS2019 and from 10.8 to 4.0% in the GS2020 (Figure 1A). Accordingly, from the DP2019, seedlings exposed to drought showed a considerable increase in cumulative mortality rate toward the experimental end (D392) from 9.1 to 35.6% (Figure 1B).

### Response of Seedling Growth to Continuous Drought

**Figure 2:**
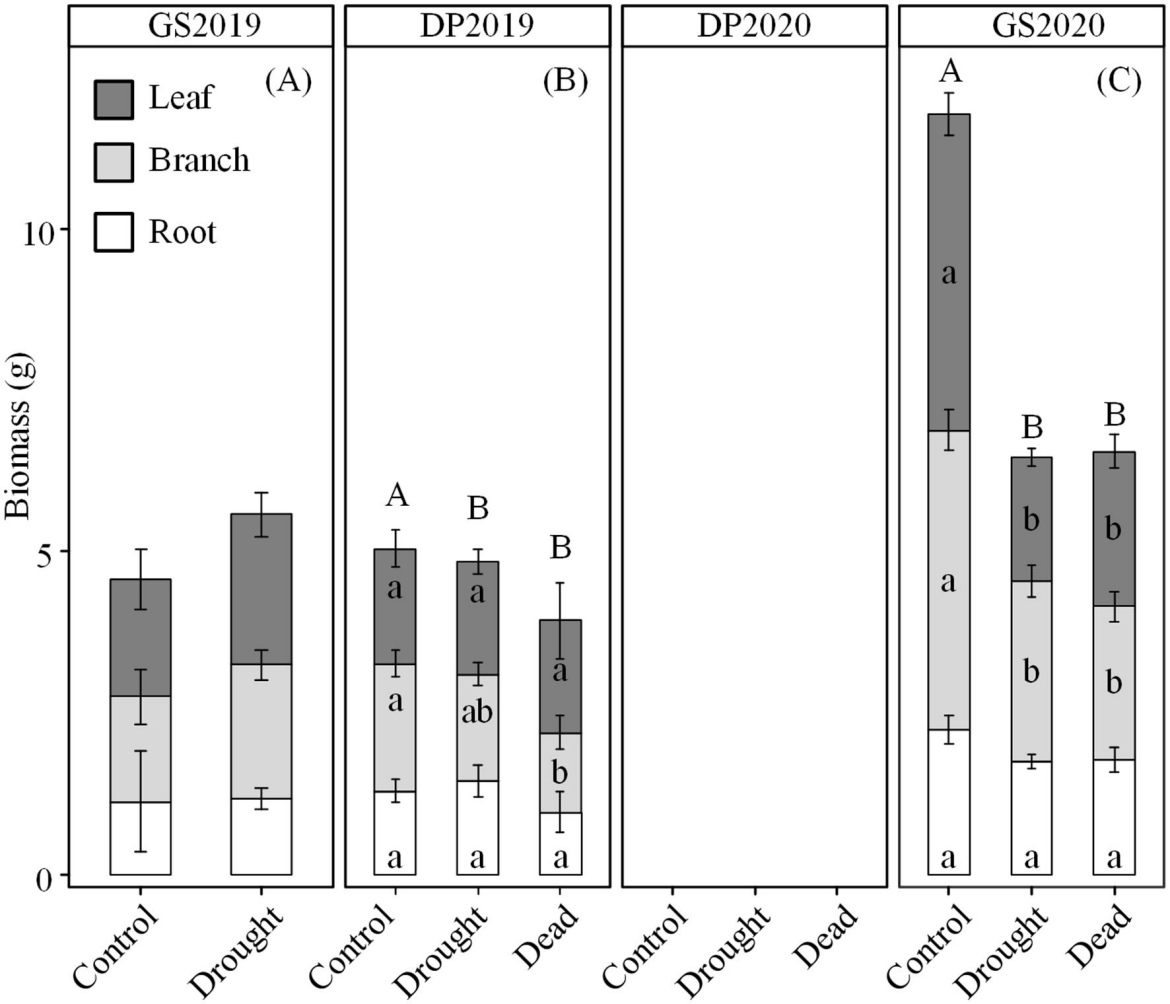
Variation in seedling biomass over the experiment period. **(A)** GS2019, the first growing season (D1–D88); **(B)** DP2019 and DP2020, the early and late winter dormant seasons (D89–D180 and D181–D301); and **(C)** GS2020, the second growing season (D302–D392). In DP2020, the biomass was not measured. Different capital letters indicate significant differences in total biomass among control, drought living, and dead seedlings on the same sampling date. Different lowercase letters indicate significant differences (*p* < 0.05) in leaf (dark gray), branch (light gray), and root (blank) among control, drought living, and dead seedlings on the same sampling date. Values are mean ± SE (*n* = 4–7).

Effects of continuous drought on seedling biomass varied significantly depending on season and organ. The whole-plant biomass was similar between control and drought treatments in the GS2019 and DP2019 (Figures 2A,B), but significantly different in the GS2020 (Figure 2C). Seedlings exposed to drought were categorized into living and dead individuals to clarify the detailed response in biomass. Branch biomass of dead but not living individuals was lower than that of seedlings in control in the DP2019 (Figure 2B). However, leaf and branch biomass of both living and dead seedlings was significantly lower than those in control in the GS2020 (Figure 2C and Supplementary Table S1). A similar trend was observed in leaf biomass in the GS2020. Root biomass had no significant variation between treatments and among organs across the experimental period.

### Response of Hydraulic Status to Continuous Drought

**Figure 3:**
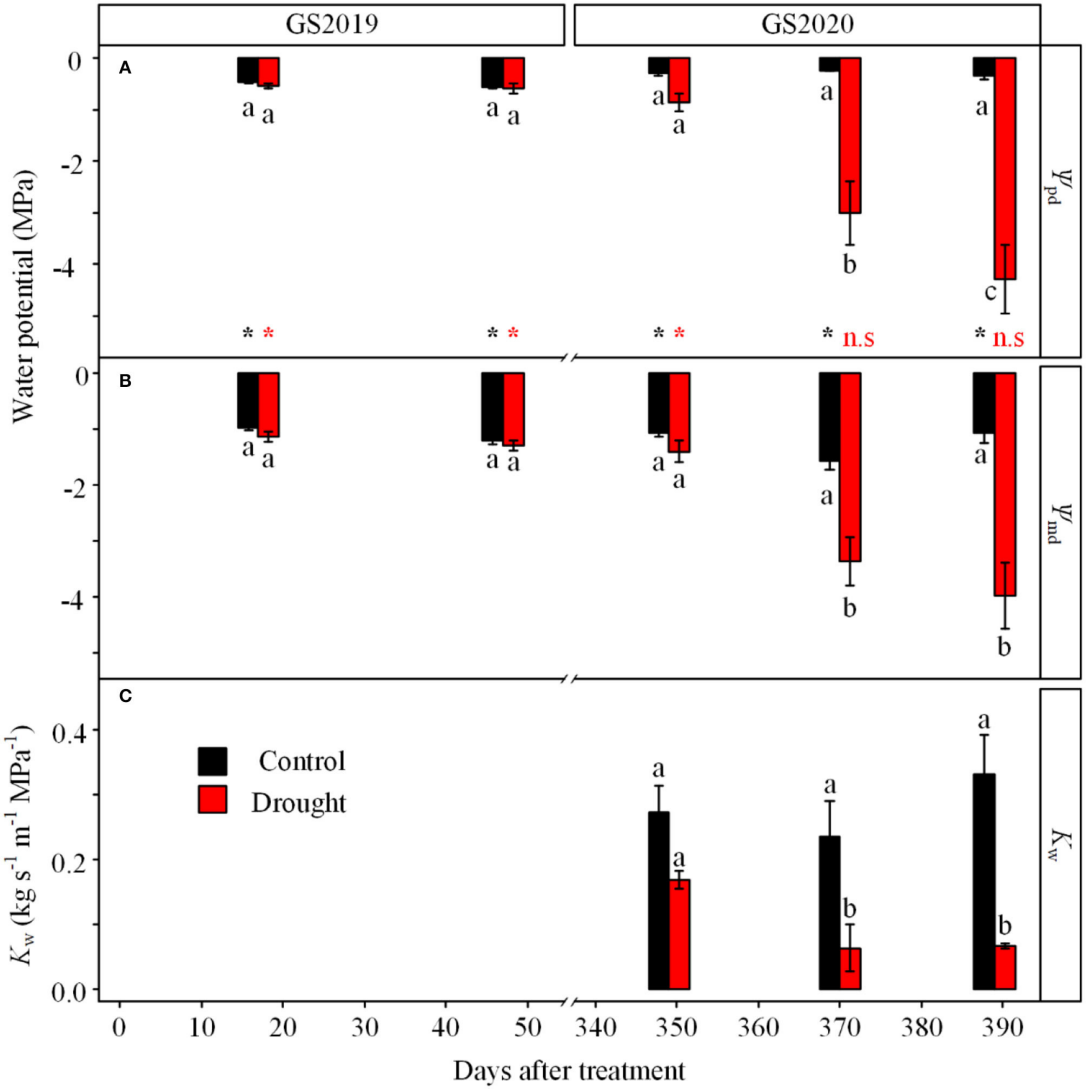
Variation in leaf water potential and stem wood-specific hydraulic conductivity (*K*_w_) in response to continuous drought. Leaf predawn water potential (ψ_pd_, **A**) and leaf midday water potential (ψ_md_, **B**) under control (black) and drought (red) treatments were measured in the GS2019 (D1–D88) and GS2020 (D302–D392). Stem wood hydraulic conductivity (*K*_w_, **C**) was measured in the GS2020. Different letters indicate significant differences among all measurement dates and between the two treatments on each measurement date (*p* < 0.05) based on the LSD test. The statistical difference between ψ_pd_ and ψ_md_ on the same measurement date is labeled with (*p < 0.05) and n.s. (not significant) at the bottom in **(A)**. Values are mean ± SE (*n* = 4–7).

Leaf water potential was significantly affected by the interaction between treatment and season (Figure 3). Leaf ψ_pd_ and ψ_md_ showed no significant difference between control and drought treatments in the GS2019 (Figures 3A,B), while both variables in seedlings exposed to drought were much lower than those to control especially in the middle and late GS2020 (Figures 3A,B). Specifically, ψ_pd_ and ψ_md_ significantly decreased from −0.9 and −1.4 MPa on D349 to −4.3 and −4.0 MPa on D390, respectively. ψ_pd_ was significantly higher than ψ_md_ in both control and drought treatments in the GS2019, while ψ_pd_ and ψ_md_ existed no significant difference under drought condition since middle GS2020 (Figures 3A,B). In terms of stem hydraulic conductivity, *K*_w_ in seedlings exposed to drought was significantly lower than those of control in the middle and late GS2020 (Figure 3C). Specifically, *K*_w_ was 27.4 and 20.2% of the control on D371 and D391, respectively (Figure 3C).

### Response of Seedling Gas Exchange to Continuous Drought

**Figure 4:**
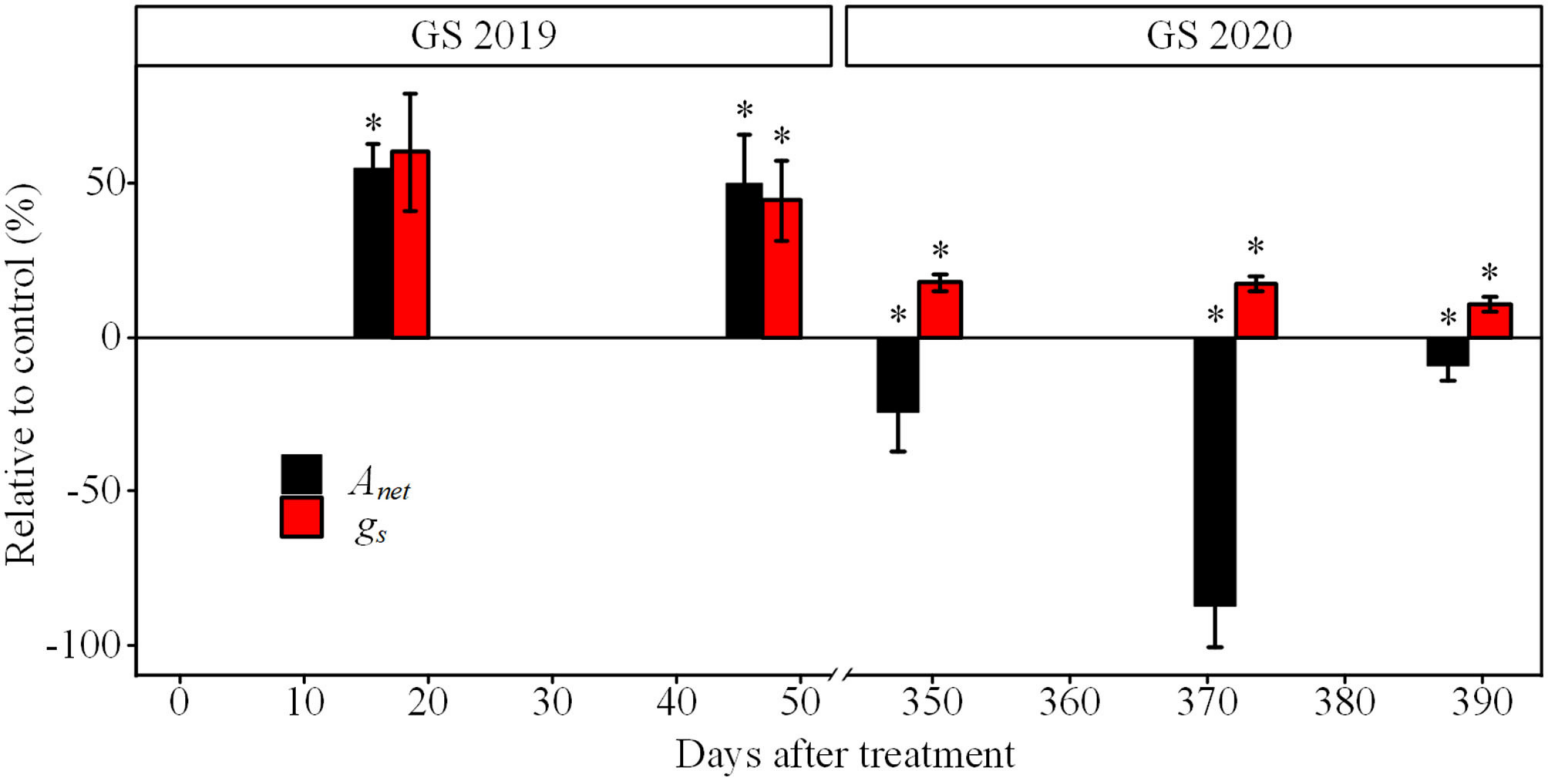
The relative difference in net photosynthetic rate (*Anet*) and stomatal conductance (*g*_*s*_) of seedlings exposed to drought and control. Asterisk indicates a significant difference between drought and corresponding control treatment on the same measurement date (*p* < 0.05) based on Wilcoxon rank-sum test. GS2019 and GS2020, the first and the second growing seasons (D1–D88 and D302–D392). Values are mean ± SE (*n* = 4–7).

Seedlings exposed to drought showed an evident decrease in *A*_*net*_ and *g*_*s*_ from the GS2019 to GS2020 (Figure 4). *A*_*net*_ was significantly lower under drought than control since D17 and accounted for 55.1% of that in control, and reached below zero in the GS2020. *g*_*s*_ under drought had a similar trend with *A*_*net*_ since D47. The *g*_*s*_ under drought decreased to 10.6% of that in control at the end of GS2020.

### Response of Seedling C Dynamic to Continuous Drought

**Figure 5:**
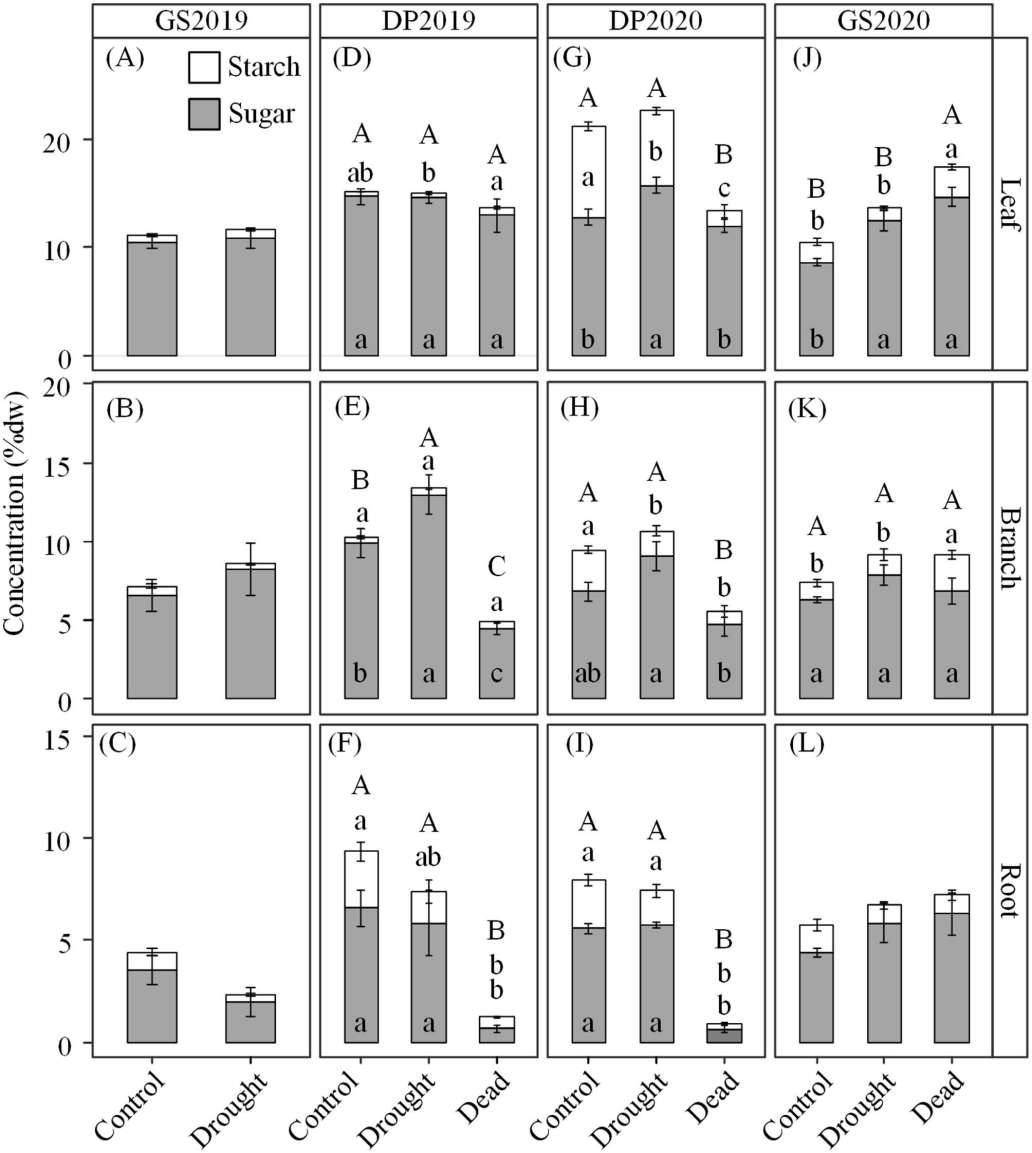
**(A–L)** Variations in concentrations of nonstructural carbohydrates (NSCs) in each organ under drought treatment over the experimental periods. GS2019, the first growing season (D1–D88); DP2019 and DP2020, the early and late winter dormant seasons (D89–D180 and D181–D301); GS2020, the second growing season (D302–D392). Different capital letters indicate significant differences (*p* < 0.05) in NSC among control, drought living, and dead seedlings. Different lowercase letters indicate significant differences (*p* < 0.05) in soluble sugars (lower) and starch (upper) among control, drought living, and dead seedlings. Gray and blank bars represent the concentrations of soluble sugars and starch, respectively. Result without significant difference was not labeled. Values are mean ± SE (*n* = 4–7).

Drought treatment, season, and their interaction significantly affected concentrations of NSC and its components, but the effect magnitude depended on organs (Table 2 and Supplementary Tables S2–S4). In two growing seasons, NSC concentrations were similar irrespective of drought treatment or organs (Figure 5). The exception was that dead individuals exposed to drought had higher NSC concentrations in leaves than control (Figure 5J). However, such response in concentrations of NSC and its components was opposite in woody organs across the winter dormant season (except for leaf in the DP2019). Especially in roots, concentrations of soluble sugars, starch, and NSC for dead individuals from drought averagely reached 0.6, 0.4, and 1.0%, respectively. Meanwhile, the concentrations of soluble sugars, starch, and NSC were 9.8, 15.4, and 11.5% of the control (Figures 5F,I).

**Table 2:** Results of two-way ANOVA for effects of season, drought, and their interaction on concentrations of soluble sugars, starch, and NSC among organs.

**Tissue**	**Factor**	***Df***	**Sugars**		**Starch**		**NSC**	
			***F***	***p***	***F***	***p***	***F***	***p***
Leaf	Season	3	**7.9**	**<0.001**	**30.7**	**<0.001**	**27.8**	**<0.001**
	Drought	2	**5.2**	**0.009**	0.4	0.839	3.1	0.054
	Season × Drought	5	**7.1**	**<0.001**	**13.4**	**0.020**	**16.1**	**<0.001**
Branch	Season	3	**3.5**	**0.023**	**21.8**	**<0.001**	1.4	0.267
	Drought	2	**15.8**	**<0.001**	1.0	0.390	**12.5**	**<0.001**
	Season × Drought	5	**3.8**	**0.006**	**8.6**	**<0.001**	**5.1**	**0.001**
Root	Season	3	7.3	0.063	**5.6**	**0.002**	6.5	0.090
	Drought	2	**7.5**	**0.024**	**27.9**	**<0.001**	**11.7**	**0.003**
	Season × Drought	5	**17.6**	**0.003**	**5.0**	**0.001**	**15.9**	**0.007**

*Bold indicates statistical significance (p < 0.05)*.

## Discussion

### Dynamic of Hydraulic and C Under Continuous Drought

The stomatal closure, one of the earliest reactions to decrease in the soil water (Figure 4), reduced the canopy water loss and maintained high water potential in the GS2019 and the early GS2020 (Figure 3). This behavior suggests a protective mechanism against embolism (McDowell et al., 2008; Xiong and Nadal, 2020), which is consistent with the finding from another spruce species (*P*. *abies*) (Hajickova et al., 2021). However, the water loss did not stop due to passive water loss and incomplete stomatal closure (Duursma et al., 2019). Moreover, water loss could be exacerbated by the relatively high temperature in the growing season (Hartmann, 2015; Yan et al., 2020). The ongoing water loss could lead to massive embolism, which constrains xylem water transport (Gebauer et al., 2020). In this study, two results could support the xylem hydraulic dysfunction in the GS2020.

First, drought significantly decreased *K*_w_ in the middle and late GS2020 (Figure 3). In this study, the *K*_w_ in living seedlings exposed to drought reduced *c*. 80% compared to the control, and complete loss of hydraulic conductivity was detected in dead seedlings. This is consistent with the previous results that severe long-term drought led to great damage in stem hydraulic integrity and hydraulic conductivity (Chen et al., 2020; Li et al., 2020).

Second, ψ_pd_ was similar to ψ_md_ in droughted seedlings since D370 (Figure 3). ψ_pd_ and ψ_md_ correspond to the daily maximum and minimum leaf water potentials, respectively (Donovan et al., 2001). ψ_pd_ recovers overnight through xylem water transport under normal water conditions (Gleason et al., 2017). Similar values of ψ_pd_ and ψ_md_ in this study suggest that massive embolism impaired water transport. Therefore, the hydraulic dysfunction might be a primary reason for mortality in the GS2020, which has been proven in field trees and potted seedlings in many extreme drought events (Adams et al., 2017; Arend et al., 2021).

Unexpectedly, living seedlings exposed to drought had similar hydraulic conductivity with control at D351 (Jun, 2020). This might indicate that the hydraulic conductance is above the critical threshold (McDowell et al., 2019) since the xylem embolism formed in the winter dormant season might be repaired (Maruta et al., 2020). It is necessary to frequently measure hydraulic dynamics after winter drought in future work.

Under continuous drought, trees may adjust phenology to maintain the C dynamic (Jin et al., 2018). In the GS2020, seedlings exposed to drought showed higher NSC but lower growth than control (Figures 2, 5). It is possible that seedlings sacrificed growth to maintain higher NSC for subsequent C use (e.g., osmoregulation) (Huang et al., 2021; Luo et al., 2021). In addition, as a drought-defoliation species, Jezo spruce fell off leaves to protect the hydraulic system, which may also stimulate C accumulation in branches and roots (Santos et al., 2022). However, C reserve may be unavailable under drought stress, and less change at the time of death (Sala et al., 2010; Jin et al., 2018; Wiley et al., 2019), which may lead to high-level NSC in drought and dead seedlings (Figure 5). Alternatively, seedlings that died in the GS2020 were collected earlier than the drought and control seedlings. The higher NSC in dead seedlings than living seedlings exposed to drought might result from an earlier occurrence of use constraint and (or) the shorter time duration of negative *A*_net_.

### Seedling Survival and C Reserve in the Winter Dormant Season

The mortality rate increased in the winter dormant season (Figure 1), suggesting that droughted seedlings might be more vulnerable in the winter dormant season. The temperature below −32°C is expected to induce frost damage to Jezo spruce (data from https://www.worldplants.ca). The minimum temperature reached −33.7°C during the experiment. Furthermore, the winter drought might aggravate the freezing damage of plants (Charrier et al., 2015; Fernández-Pérez et al., 2018).

Nonstructural carbohydrate (NSC) plays a critical role in drought and cold tolerance of temperate trees (Charrier et al., 2015). In contrast to the growing season, we detected NSC depletion in the roots of dead seedlings in the winter dormant season (Figure 5). This is consistent with the results of Galvez et al. (2013), which showed that insufficient NSC reserve in roots might be the primary reason for seedling mortality of two poplar species in the winter dormant season. However, the concentrations of soluble sugars and starch did not decrease to zero in dead seedlings, indicating that there may be a minimum threshold of NSC reserve for tree survival (McDowell, 2011). In this work, NSC concentration in dead seedlings is consistent with the life-threatening NSC level in previous drought studies (Schönbeck et al., 2020), defoliation (Barker Plotkin et al., 2021), and shade (Weber et al., 2018, 2019).

In this study, drought (living) seedlings had higher or similar soluble sugars than control (Figure 5). Similar results were also observed in previous studies, e.g., Chuste et al. (2020) and Schönbeck et al. (2020). These support the theory that NSC concentration initially increases and shifts to decrease with drought persisting (McDowell, 2011). It is possible that NSC reserve might increase or be stable during the early drought stage by limiting growth respiration and consuming with drought persisting (McDowell, 2011). In this work, different death times among seedlings under drought treatment might indicate the different exact physiological stress stages among seedlings (Zhang et al., 2021). However, the reason why seedlings exposed to the same drought showed different death times should be further investigated.

The insufficient NSC reserve and severe embolism may threaten tree survival in the winter (Galvez et al., 2013). Since the lack of hydraulic conductivity and PLC data in the winter dormant season, it is unable to assess the direct contribution of hydraulic dysfunction to seedling mortality during the dormant season. However, almost complete loss of hydraulic conductance may not be damaging due to stomatal closure and reduction in water uptake (Beikircher et al., 2016). As shown in previous studies (Ogasa et al., 2019; Mayr et al., 2020), trees that experienced near to 100% PLC in winter recovered growth in the following seasons, suggesting the whole tree and tissues were still alive in the winter dormant season. Thus, future studies are required to explore the appropriate methods for assessing the linkage between tree mortality and hydraulic dysfunction in the winter dormant season under drought stress.

## Conclusion

This study demonstrated that seedlings subjected to continuous drought showed a significant decrease in net photosynthesis rate, stomatal conductance, and biomass in the second than the first growing season, while the seedling mortality continually increased toward the end of the second growing season. Under drought stress, seedlings with root concentrations of both soluble sugars and starch less than 1% died in the winter dormant season. Hydraulic conductivity was significantly lost in the growing season. These suggest that C starvation may partly explain seedling mortality in the winter dormant season, while hydraulic failure may determine seedling survival in following growing seasons with continuous drought. Two processes may interactively cause more tree death in northern temperate forests in the case that the drought duration is projected to extend associated with climate change in the future.

## Data Availability Statement

The original contributions presented in the study are included in the article/Supplementary Material, further inquiries can be directed to the corresponding authors.

## Author Contributions

DY and YH made the experimental design. YH conducted the experiment and drafted the manuscript. JD, Q-WW, and WZ improved the manuscript draft. DY and Q-WW revised and finalized the manuscript. All authors contributed to manuscript completion and revision.

## Funding

This work was supported by the National Natural Science Foundation of China (41871105 and 41971148).

## Conflict of Interest

The authors declare that the research was conducted in the absence of any commercial or financial relationships that could be construed as a potential conflict of interest.

## Publisher's Note

All claims expressed in this article are solely those of the authors and do not necessarily represent those of their affiliated organizations, or those of the publisher, the editors and the reviewers. Any product that may be evaluated in this article, or claim that may be made by its manufacturer, is not guaranteed or endorsed by the publisher.
