# Supplementary material for: Seasonal Responses of Hydraulic Function and Carbon Dynamics in Spruce Seedlings to Continuous Drought
Source: Front Plant Sci. 2022 May 4;13:868108. doi: 10.3389/fpls.2022.868108 (PMC9115555; doi:10.3389/fpls.2022.868108)
Supplement: Supplementary file 1 [file Presentation_1.pdf]

Seasonal responses of hydraulic function and carbon dynamics in spruce seedlings to continuous drought

Yangang Han[1,2], Jiaojiao Deng[1], Wangming Zhou[1], Qing-Wei Wang[1*], Dapao Yu[1*]

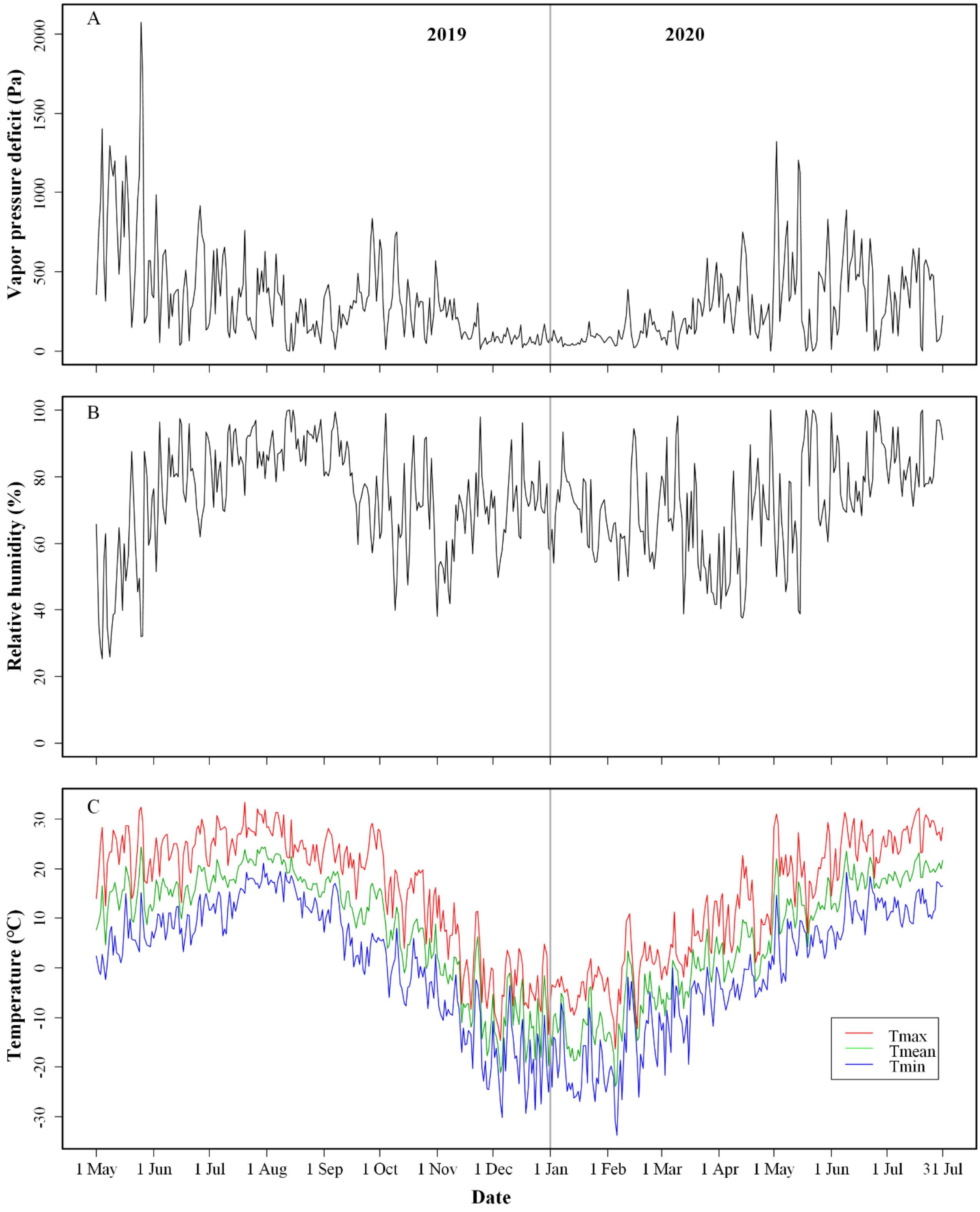

Figure S1 Diurnal dynamics of a) vapor pressure deficit (VPD), b) relative humidity, c) temperature. Vertical lines indicate the first of the year 2020.

Table S1 Dynamic of leaf biomass during different seasons. Different letters indicate significant difference (p<0.05) between different treatments and different seasons. Values are mean ± SE.

Season	Treatment	Leaf
GS2019	Control	1.8143±0.4710 b
	Drought	2.3236±0.3374 b
DP2019	Control	1.7709±0.2866 b
	Drought	1.7479±0.1923 b
	Dead	1.7349±0.5894 b
GS2020	Control	4.8848±0.332 a
	Drought	1.9178±0.1435 b
	Dead	2.4025±0.2556 b

Table S2 Concentrations of soluble, starch and NSC among different seasons in leaf. Different letters in the same column indicate significant difference among different seasons. Values are mean ± SE.

Season	Soluble sugars			Starch			NSC		
	Control	Drought	Dead	Control	Drought	Dead	Control	Drought	Dead
GS2019	10.45±·0.57 b	10.91±0.94 c		0.72±0.09 c	0.71±0.06 bc		11.17±0.58 c	11.62±0.93 c	
DP2019	14.69±0.72 a	14.58±0.56 ab	12.94±1.58 a	0.47±0.04 c	0.41±0.12 c	0.76±0.12 b	15.16±0.72 b	14.99±0.64 b	13.7±1.65 b
DP2020	12.79±0.79 a	15.70±0.73 a	11.96±0.60 a	8.38±0.44 a	6.87±0.30 a	1.41±0.61 b	21.16±1.18 a	22.57±0.86 a	13.37±0.82 b
GS2020	8.63±0.29 c	12.50±0.95 bc	14.66±0.91 a	1.85±0.36 b	1.12±0.15 b	2.73±0.23 a	10.48±0.53 c	13.63±1.09 bc	17.39±1.06 a

Table S3 Concentrations of soluble, starch and NSC among different seasons in branch. Different letters in the same column indicate significant difference among different seasons. Values are mean ± SE.

Season	Soluble sugars			Starch			NSC		
	Control	Drought	Dead	Control	Drought	Dead	Control	Drought	Dead
GS2019	6.56±1.01 b	8.27±1.66 b		0.61±0.11 bc	0.31±0.05 b		7.17±1.04 b	8.57±1.69 b	
DP2019	9.89±0.89 a	12.96±1.23 a	4.46±0.36 a	0.37±0.10 c	0.40±0.07 b	0.43±0.07 b	10.26±0.97 a	13.37±1.16 a	4.89±0.31 b
DP2020	6.82±0.57 b	9.05±0.92 b	4.77±0.78 a	2.64±0.22 a	1.63±0.29 a	0.81±0.38 b	9.46±0.52 ab	10.67±0.76 ab	5.59±0.70 b
GS2020	6.34±0.19 b	7.87±0.61 b	6.87±0.84 a	1.04±0.21 b	1.28±0.36 a	2.31±0.26 a	7.37±0.22 b	9.16±0.90 b	9.18±1.05 a

Table S4 Concentrations of soluble, starch and NSC among different seasons in root. Different letters in the same column indicate significant difference among different seasons. Values are mean ± SE.

Season	Soluble sugars			Starch			NSC		
	Control	Drought	Dead	Control	Drought	Dead	Control	Drought	Dead
GS2019	3.53±0.73 c	1.97±0.69 b		0.87±0.18 b	0.36±0.09 b		4.40±0.85 c	2.33±0.77 b	
DP2019	6.57±0.88 a	5.83±1.63 a	0.65±0.15 b	2.76±0.46 a	1.53±0.6 a	0.58±0.03 ab	9.34±1.09 a	7.36±2.22 a	1.23±0.14 b
DP2020	5.55±0.28 ab	5.71±0.13 a	0.62±0.18 b	2.37±0.26 a	1.72±0.32 a	0.29±0.05 b	7.92±0.20 ab	7.43±0.30 a	0.91±0.17 b
GS2020	4.38±0.24 bc	5.83±0.94 a	6.28±1.03 a	1.36±0.29 b	0.88±0.17 ab	0.93±0.25 a	5.75±0.49 bc	6.71±1.09 a	7.21±1.19 a
